# NADPH Oxidase (NOX) Targeting in Diabetes: A Special Emphasis on Pancreatic β-Cell Dysfunction

**DOI:** 10.3390/cells10071573

**Published:** 2021-06-22

**Authors:** Suma Elumalai, Udayakumar Karunakaran, Jun-Sung Moon, Kyu-Chang Won

**Affiliations:** 1 Innovative Center for Aging Research, Yeungnam University Medical Center, Daegu 42415, Korea; sumalakshmi@gmail.com (S.E.); udayactech@gmail.com (U.K.); 2 Department of Internal Medicine, Yeungnam Universtiy College of Medicine, Daegu 42415, Korea

**Keywords:** NADPH oxidase, diabetes mellitus, insulin-secreting cells, oxidative stress, reactive oxygen species

## Abstract

In type 2 diabetes, metabolic stress has a negative impact on pancreatic β-cell function and survival (T2D). Although the pathogenesis of metabolic stress is complex, an imbalance in redox homeostasis causes abnormal tissue damage and β-cell death due to low endogenous antioxidant expression levels in β-cells. Under diabetogenic conditions, the susceptibility of β-cells to oxidative damage by NADPH oxidase has been related to contributing to β-cell dysfunction. Here, we consider recent insights into how the redox response becomes deregulated under diabetic conditions by NADPH oxidase, as well as the therapeutic benefits of NOX inhibitors, which may provide clues for understanding the pathomechanisms and developing strategies aimed at the treatment or prevention of metabolic stress associated with β-cell failure.

## 1. Introduction

Type 2 diabetes (T2D) is a complicated metabolic condition marked by peripheral insulin resistance [1], obesity [1], hyperglycemia [2], and elevated levels of cytokines [3], all of which contribute to a lack of insulin and consequent β-cell failure. Further, considerable evidence has been presented that explains this metabolic dysfunction, including evidence that indicates the role of mitochondrial dysfunction, oxidative stress, endoplasmic reticulum (ER) stress, hyperglycemia (glucotoxicity), dyslipidemia, and the combination of both, glucolipotoxicity [4]. The activation of chronic inflammation is another way that this diabetogenic environment leads to the onset and/or progression of type 2 diabetes. Furthermore, there is strong evidence that suggests that chronically elevated levels of reactive oxygen species (ROS) lead to increased oxidative stress in β-cells. Given the ability of ROS to directly damage and oxidize DNA, proteins, and lipids, β-cell functioning is worsened in terms of insulin secretion and action [5,6]. NADPH oxidase (NOX) proteins are membrane-associated multiunit enzymes that play a physiological role in response to various factors, as well as pathophysiological roles in diabetic pancreatic β-cells. In this review of the current literature, we focus on the role of NOX enzymes in signal transduction in pancreatic β-cells, as well as discuss how these cells might contribute to the development of type 2 diabetes.

## 2. NADPH Oxidase Isoforms: An Overview

**Figure 1:**
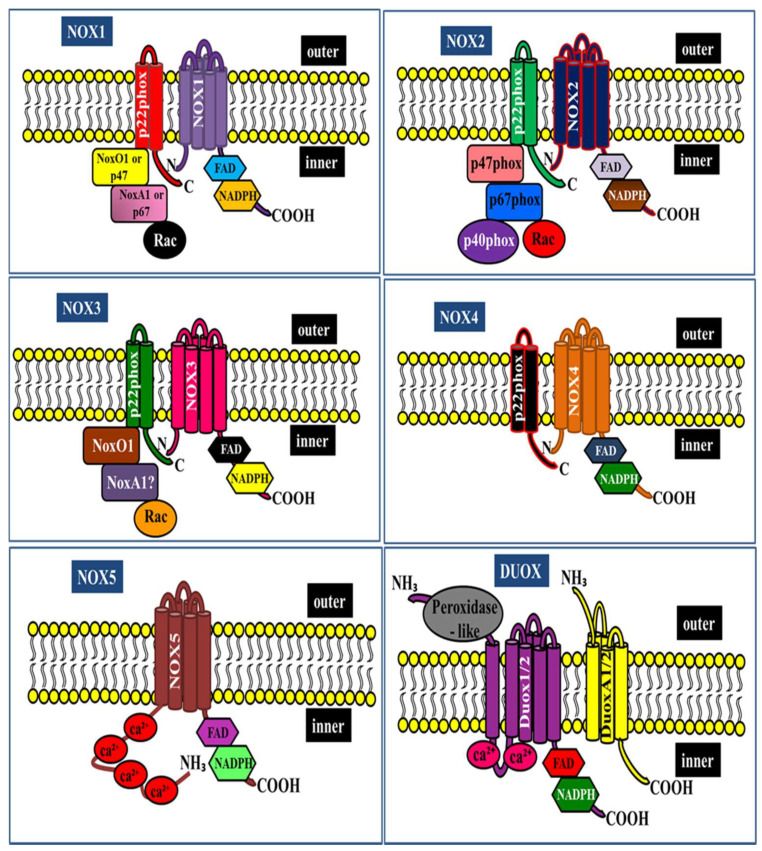
NADPH oxidase family structure and its subunits.

NOX enzymes are multi-subunit enzymes that transfer electrons across biological membranes and are essential for superoxide production. The prototypical NADPH oxidase NOX2, which catalyzes the NADPH-dependent reduction of oxygen to superoxide in phagocytes, was the first enzyme discovered [7]. The phagocytic NOX2 comprises six different subunits that interact to form an active enzyme complex. The catalytic gp91phox and p22phox subunits are located in the plasma membrane, and the regulatory (p47phox, p67phox, p40phox, and small G-protein Rac1/2) subunits are present in the cytosolic components and assembly of all NOX components responsible for the production of superoxide [8,9]. Under resting conditions, the multi-subunit enzyme is inactive. Upon stimulation, the entire cytosolic regulatory complex subsequently translocates to the plasma membrane and associates with catalytic subunits to form the active oxidase, generating superoxide by transferring an electron from NADPH in the cytosol to oxygen on the luminal space [10,11]. In parallel with the progress toward understanding the phagocytic NOX, a series of studies have identified six NOX2 homologs (NOX1, NOX3, NOX4, NOX5, DUOX (dual-oxidase) 1, and DUOX2) in mammalian systems [12,13,14,15]. There are indeed significant similarities and differences between the NOX homologs, for example, subunits that bind NOX1 are NOXO1 (NOX organizer 1) and NOXA1 (NOX activator 1) [16]. Notably, all members of the NOX family contain at least six transmembrane domains, and cytosolic FAD and NADPH-binding domains. However, NOX5 and DUOX1/2 contain extra functional domains that lack NOX1–4. NOX5 contains EF-hand Ca^2+^-binding domains, whereas DUXO1/2 has a seventh transmembrane domain at the NH2 terminus with an extracellular peroxidase-like domain in addition to the EF-hand and gp91phox homology domains [17,18] (Figure 1).

## 3. The Role of NADPH Oxidase in Insulin Secretion

Pancreatic β-cells are specialized endocrine cells that secrete insulin to maintain normal fuel homeostasis in response to nutrients, as well as hormonal, neural, and pharmacological factors. Among these, glucose is the most important regulator of insulin secretion via the glycolytic and respiratory metabolism-mediated generation of reactive oxygen species (ROS), leading to accelerated ATP generation in β-cells. The plasmalemmal ATP-sensitive potassium channel (KATP) closes as the ATP:ADP ratio in the cytoplasm rises, resulting in plasma membrane depolarization and the opening of the voltage-gated Ca^2+^ channel. This causes an influx of Ca^2+^ into the cell, which causes insulin granules to exocytose [19,20]. Importantly, increasing evidence suggests that brief high-glucose exposure to isolated mouse islets and rat INS-1 (832/13) cells resulted in a significant accumulation of intracellular H_2_O_2_ at the same time as increased glucose-stimulated insulin secretion (GSIS) with decreased GSH-to-GSSG ratios. The ratio of GSH to GSSG has long been thought to be a good indication of oxidative stress and redox signaling. As a result, the observed drop in the GSH-to-GSSG ratio in response to glucose stimulation is consistent with the formation of ROS following high glucose, and could indicate the quenching of the H_2_O_2_ signal. Furthermore, H_2_O_2_-stimulated insulin secretion is a Ca^2+^-dependent extracellular activity, implying that H_2_O_2_ is involved in Ca^2+^ influx [21,22]. Although mitochondria remain the primary source of free radicals, emerging evidence implicates NOX as a major source of mitochondrial ROS in β-cells. Furthermore, there is considerable evidence regarding the expression of NOX1, NOX2, NOX4, and p22phox, as well as cytosolic regulators p40phox, p47phox, and p67phox, and their homologs Noxo1 and Noxa1 in pancreatic β-cells [23,24,25]. However, the pharmacological inhibition of NOX using diphenyleneiodonium, a non-selective flavoprotein inhibitor, impairs GSIS, which is attributed to the importance of hydrogen peroxide production in beta cells [24,26]. However, this drug also inhibits complex I of the electron transport chain and K^+^ current via a mechanism that does not involve the inhibition of H_2_O_2_ production by a NOX enzyme. In addition, the Ca^2+^ current might be attributed to poor specificity, thereby misleading the function of NOXs in β-cells [27]. In pancreatic rat islets, it was found that antisense blockage of p47phox subunits markedly reduced the stimulatory glucose-induced H_2_O_2_ production and GSIS, thus avoiding the poor specificity of NOX inhibitors [28]. In contrast, the individual deletion of NOX isoforms did not impair glucose-stimulated insulin secretion in mouse islets. Moreover, NOX2-null islets exhibited enhanced glucose-stimulated secretory responses through the reduction in ROS generation. In addition, NOX2-deficient islets showed elevated cAMP levels, suggesting that cAMP potentiates the secretory response. There is evidence regarding the inhibition of p47phox phosphorylation through the cAMP-mediated reduction in superoxide production and via the blocking of the respiratory burst in neutrophils [29]. Phosphodiesterase, the predominant enzyme that selectively hydrolyzes and inactivates cAMP, is present in the pancreatic beta cells and plays a role in insulin secretion [30]. In addition, the inhibition of phosphodiesterase has been shown to reverse the NADPH catalytic subunit gp91phox-mediated, oxidative stress-induced behavioral signature, that is, depression and anxiety by cAMP signaling [31]. However, the downstream mechanism by which NOX2-deficient islets lead to cAMP activation is unknown. With this functional characterization, we suggest that the phosphodiesterase–NOX2 axis may contribute to defects in cAMP activation as well as insulin secretion. Further investigations are needed to clarify these unexpected findings.

## 4. The Role of NADPH Oxidase in Hyperglycemia-Induced β-Cell Dysfunction

Chronic hyperglycemia has deleterious effects on pancreatic β-cell function and survival in patients with type 2 diabetes (T2D). Hyperglycemia stimulates increased glycolytic flux and the subsequent production of reducing equivalents, leading to the production of ROS, including superoxide, hydrogen peroxide, and hydroxyl radicals [32,33,34]. Recent studies have shown that superoxide radicals generated by NOXs could lead to both the induction of intracellular oxidative stress in pancreatic β-cell damage and apoptosis [25,35,36]. Several pieces of evidence support a key role of RAC1, a small guanosine triphosphate (GTP)-bound protein required for the assembly and catalytic activation of NOX2 [37,38,39]. In addition, RAC1 has long been recognized as a signaling molecule in cytoskeletal dynamics and in a variety of cellular processes, including cell polarization, morphogenesis, migration, apoptosis, vesicle trafficking, and cellular transformation [40]. Furthermore, pancreatic β-cell-specific RAC1 deficiency causes the polymerization of F-actin, which restricts the recruitment of insulin granules at the surface of the plasma membrane, leading to reduced insulin secretion [41]. However, guanine–nucleotide exchange factors facilitate the detachment of guanosine diphosphate, and the binding of GTP may control the conversion of inactive RAC1 to the active RAC1-GTP form [42,43]. Several guanine exchange factors (T-cell lymphoma invasion and metastasis (TIAM1), the oncogene F proto-oncogene (VAV2), and the triple functional domain protein (Trio)) are known to mediate RAC1 activation [44]. Activated RAC1 recruits p67phox, which associates with p47phox and increases superoxide production [45,46]. On the other hand, evidence suggests that TIAM1 triggers RAC1 activation to increase the dysfunction and apoptosis of pancreatic beta cells under high glucose stress via the activation of NOX. Moreover, it should be noted that the activation of JNK signaling lies upstream of mitochondrial dysfunction and caspase-3 activation in response to high glucose [36]. Therefore, it is likely that the inhibition of RAC1 activation reduces NOX-mediated oxidative stress and ROS-mediated JNK signal transduction. An additional consideration is how changes in the balance of antioxidant enzymes, and increases in ROS production by mitochondria, can alter the susceptibility to dysfunction by the activation of stress kinases. In addition, under hyperglycemic conditions, elevated levels of diacylglycerol activate PKC, which subsequently increases the levels of oxidants, such as H_2_O_2_ via the PKC-dependent activation of NOX [47]. Furthermore, mice deficient in NOX2 were shown to have attenuated β-cell destruction and preserved islet function in streptozotocin-induced diabetes, partially through the reduction in ROS generation [48]. In contrast, NOX2 deficiency increased GSIS and did not prevent high glucose-induced islet DNA fragmentation and β-cell apoptosis [49]. Alternatively, the pharmacological inhibition of NOX1–4 protected against the high glucose-induced inhibition of human islet insulin release and survival [50,51]. The reason for this inconsistency is unclear. Thus, NOX-induced oxidative stress and mitochondrial dysfunction contribute to impaired endogenous antioxidant defenses, leading to pancreatic β-cell dysfunction in diabetes.

An additional consideration is the upregulation of NOX enzymes in response to chronic hyperglycemia. This activates the angiotensin II type 1 receptor (AT1R) and increases superoxide production, as well as p47phox and p22phox expression in a rat insulin-producing cell line and human pancreatic islets [52,53]. Furthermore, hyperglycemia and angiotensin II type 1 receptor-induced proinflammatory cytokines in human islets cause impaired insulin secretion and inflammation [54,55]. The inhibition of AT1R selectively downregulates NOX; this, in turn, suppresses oxidative stress, thus improving β-cell insulin secretion and decreasing β-cell apoptosis [56]. Indeed, it has been observed that proinflammatory cytokines upregulate NOX1 in β-cells, leading to the loss of function and activation of oxidative stress linked to β-cell failure [57,58]. In addition, pro-inflammatory cytokines induce 12-lipoxygenase (12-LO) expression, which may be a mediator of NOX1-induced β-cell dysfunction by producing 12-hydroxyeicosatetraenoic acid from arachidonic acid. In addition, the inhibition of 12-LO activity blocked the induction of NOX1 by pro-inflammatory cytokines [59,60]. On the other hand, there is increasing evidence that cytokines activate RAC1, which subsequently increases the levels of oxidants via the activation of NOX2. This contributes to alterations in mitochondrial function, leading to caspase-3 activation and the metabolic dysfunction of β-cells. The inhibition of RAC1 activation significantly suppressed the cytokine-induced activation of iNOS and NOX2 activation and the alterations in downstream signaling events involved in cell dysfunction [61,62,63,64]. These results help integrate intracellular events with an elevation of ROS stimulated by pro-inflammatory cytokines, culminating in the onset of islet dysfunction and diabetes. Further investigation is needed to clarify these findings.

## 5. The Role of NADPH Oxidase in β-Cell Dysfunction Caused by Lipotoxicity

**Figure 2:**
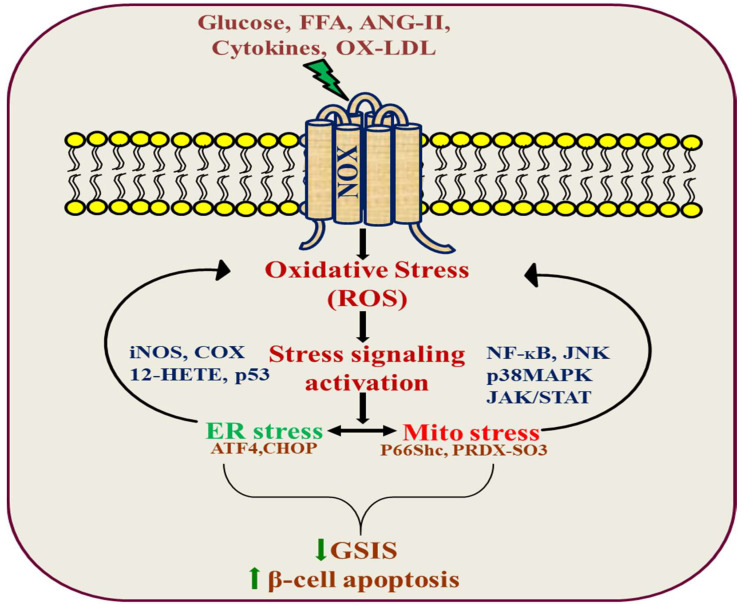
NADPH oxidase signal transduction in pancreatic β-cell dysfunction.

Several studies have shown that elevated levels of glucose, along with circulating free fatty acids (FFAs), constitute the major causes of insulin resistance and β-cell dysfunction during the development of type 2 diabetes [65,66,67,68]. The exposure of β-cells to high glucose induces an increased expression of CD36 in the plasma membrane, and may exacerbate disease aggravation over time by inducing fatty acid uptake, leading to metabolic and functional alterations [69,70]. Interestingly, this effect was mediated by a robust upregulation of the RAC1-mediated activation of NOX activity. Furthermore, the inhibition of RAC1-NOX activation blocked high glucose-induced CD36 expression as well as fatty acid uptake and the downstream signal transduction-mediated cell apoptosis [71]. Moreover, CD36 deficiency attenuates obesity-associated oxidative stress in the heart by reducing NOX activity [72]. Although evidence of direct interaction between NOX and CD36 is still lacking, we found that inhibitors of each target can abrogate the downstream signaling damage in response to high glucose [71,73]. CD36 modulates cellular FFA transfer, and studies with the Zucker diabetic fatty (ZDF) rat, an obesity-induced diabetic animal model, indicate that elevated levels of FFA cause ceramide accumulation, which destroys β-cells by apoptosis. In addition, the increased activation of RAC1 and NOX-associated oxidative stress, in turn, promotes the activation of JNK1/2 and mitochondrial dysregulation in ZDF rats [36]. Based on the existing information, we also found that ceramide-induced β-cell functional defects, through the engagement of Rac1-NOX signaling, coordinates JNK activation, contributing to insulin resistance, obesity, and the production of inflammatory cytokines [73,74,75,76]. However, the mechanism by which the NOX-JNK interlink promotes β-cell damage is not completely understood. It was recently shown that p66Shc mediates lipotoxicity-induced apoptosis in pancreatic β-cells, suggesting that p66Shc could sense the impaired metabolic changes in diabetes and promote cellular dysfunction [77]. p66Shc is a member of the ShcA family of adaptor proteins that consists of three members: p46Shc, p52Shc, and p66Shc, which are derived from the same transcript via an alternative splicing of two distinct ATG start codons. p52/p46Shc contains three phospho-tyrosine sites at Y239, Y240, and Y317. These are located in the CH1 region, enabling Shc proteins to recruit the Grb2-SOS complex that activates the GTPase Ras and the Grb2-Gab2-phosphoinositide-3-kinase (PI3K) complex, which is necessary for transmitting mitogenic and cell survival signals to downstream targets. Furthermore, the longest isoform, p66Shc, possesses an additional *N*-terminal CH2 region of 110 amino acids, containing an S36 phosphorylation site that has been implicated in mediating oxidative stress signaling [78,79,80]. Earlier studies have suggested that JNK-dependent p66Shc serine36 phosphorylation leads to ROS production and cell death [81]. Based on these results, we found that JNK-dependent p66Shc serine36 phosphorylation leads to ROS production and cell death by ceramide in a NOX-dependent manner [73]. These signaling events subsequently promote the translocation of p66Shc to mitochondria to drive reactive oxygen production and promote the activation of oxidized PRDX3 accumulation in the mitochondria, which would favor MPTP opening and mitochondrial swelling [73]. These observations were further supported by using NOX2 knockout mice, suggesting that NOX2-dependent H_2_O_2_ production is a likely cause of early palmitate-dependent impairment in insulin secretion and the induction of β-cell dysfunction [82]. However, experimental data suggest that palmitate triggers transient receptor potential melastatin (TRPM)-2 channels by activating NOX2. The induction of TRPM2 channels caused an increase in mitochondrial Zn^2+^, leading to mitochondrial membrane potential loss and mitochondrial fission [83]. Further understanding of this link should provide valuable insights for the identification of therapeutic targets to protect β-cell function and prevent T2D. It has also been demonstrated that oxidized LDL induces β-cell dysfunction via reactive oxygen species and radical lipid hydroperoxides [84,85]. However, it is not clear how OX-LDL regulates apoptotic stimuli in beta cells. Importantly, OX-LDL induces oxidative stress in neutrophils by activating NOX via TLR-PKC-IRAK-MAPK [86]. However, the downstream mechanism by which OX-LDL leads to apoptosis is not clear, and this may be because the crosslink between OX-LDL and NOX may determine cellular commitment to apoptosis through ROS production. In addition, ER stress is linked to oxidative stress in oxidized LDL-induced β-cell dysfunction and death [87]. There is also a connection between OX-LDL and ER stress via a robust upregulation of NOX4 in human umbilical vein endothelial cells [88,89,90]. Together, these findings suggest the possibility of a novel pathway involving the ER and mitochondria, through which these organelles orchestrate the regulation of death signals. Further understanding of this link should provide insights for the identification of therapeutic targets to protect β-cell function and prevent T2D (Figure 2).

## 6. NADPH Oxidase-Targeted Therapeutic Agents

NOX is associated with β-cell dysfunction, apoptosis, insulin resistance, and T2D. Therefore, agents that reduce NOX expression or activity are useful in treating obesity, peripheral insulin resistance, hyperglycemia, and T2D. The chemical inhibitor apocynin (4’-hydroxy-30methoxyacetophenone), a naturally occurring methoxy-substituted catechol, and DP1 diphenyleneiodonium, a non-selective flavoprotein inhibitor, are considered selective NOX inhibitors [24,26,36,48,61]. Importantly, these compounds have off-target effects and are not considered as selective NOX inhibitors because their specificity of action has been questioned in previous reports [91,92]. However, two nontoxic, pyrazolopyridine dione-based, structurally related compounds, GKT136901 and GKT137831, demonstrating a preferential inhibition of NOX1 and NOX4, have been identified and proposed as a novel therapy for NOX inhibition [93,94]. In addition, active phenothiazine (2-acetylphenothiazine) compounds have been shown to inhibit NOX1, and cell-permeable thiotriazolopyrimidine compounds are promising candidates for both exploring the role of NOX subunits in disease pathology, and to assess their inhibitory activity as a new therapeutic approach to disease [95,96,97]. There is a growing body of evidence that metformin ameliorates lipotoxic dysfunction by inhibiting oxidative stress and ER stress mediated by NOX in β-cells, as well as in other experimental diabetes models [98,99,100]. Furthermore, the activation of PPARγ by pioglitazone, a thiazolidinedione class of antidiabetic agents, was shown to inhibit ANG II-induced COX-2 expression, likely by interfering with downregulating ROS production via inhibiting NOX activity [101]. Recently, a novel DPP-4 inhibitor, teneligliptin, was found to exhibit a broad spectrum of bioactivities, alter the pro-inflammatory phenotype of adipocytes, and inhibit atherogenesis by reducing the expression of NOX4, a major NOX subunit in adipocytes [102]. Similarly, we found that teneligliptin treatment suppressed high glucose-mediated β-cell oxidative stress by increasing the stability and activity of SIRT1 [103]. In addition, it has been reported that the upregulation of NOX oxidase subunits p22phox and NOX4 eventually leads to endothelial dysfunction due to superoxide production via SIRT1 inhibition [104]. Plant-derived flavonoids have recently been discovered to have a diverse range of biochemical and pharmacological activities that may have a major impact on the functions of various cellular systems [105,106]. In this context, we have also shown that myricetin (3, 5, 7, 3′, 4′, 5′-hexahydroxyflavone) enhances β-cell function and survival after thapsigargin-induced endoplasmic reticulum stress via NOX inactivation [107]. Based on the results of these studies, we can conclude that compounds that inhibit the NOX subunits show promise as a means of exploring the role of NOX subunits in disease pathology, and that the assessment of their inhibition could potentially lead to the development of novel therapeutics to prevent T2D.

## 7. Conclusions

There is experimental evidence of the contribution of NOX to pancreatic β-cell dysfunction under diabetogenic conditions. Understanding this complex scenario and the function of NOX-activated redox-regulated pathways may lead to the improved treatment of β-cell failure and T2DM; however, due to low specificity and off-target effects, answers to several key questions remain elusive. Thus, isoform-specific inhibitors, or genetic models in pancreatic β-cells, are required to determine the link between NOX and β-cell failure. This could contribute to the development of novel therapeutics for the prevention and treatment of T2DM.

## Data Availability

Not applicable.
